# Plasma Cytokine Profile in Tropical Endomyocardial Fibrosis: Predominance of TNF-a, IL-4 and IL-10

**DOI:** 10.1371/journal.pone.0108984

**Published:** 2014-10-10

**Authors:** Aline S. Bossa, Vera M. C. Salemi, Susan P. Ribeiro, Daniela S. Rosa, Ludmila Rodrigues Pinto Ferreira, Suzete C. Ferreira, Anna Shoko Nishiya, Charles Mady, Jorge Kalil, Edecio Cunha-Neto

**Affiliations:** 1 Laboratory of Immunology, Heart Institute (InCor), University of São Paulo School of Medicine, São Paulo, Brazil; 2 Division of Clinical Immunology and Allergy, University of São Paulo School of Medicine, São Paulo, Brazil; 3 Cardiomyopathy Unit, Heart Institute (InCor), University of São Paulo School of Medicine, São Paulo, Brazil; 4 Pró-Sangue Foundation, São Paulo, Brazil; Institute for Investigation in Immunology (iii), INCT, São Paulo, Brazil; 5 Division of Immunology, Department of Microbiology, Immunology and Parasitology-Federal University of São Paulo-UNIFESP, São Paulo, Brazil; University of Montreal Hospital Research Center (CRCHUM), Canada

## Abstract

**Background:**

The participation of immune/inflammatory mechanisms in the pathogenesis of tropical endomyocardial fibrosis (EMF) has been suggested by the finding of early blood and myocardial eosinophilia. However, the inflammatory activation status of late-stage EMF patients is still unknown.

**Methodology/Principal findings:**

We evaluated pro- and anti-inflammatory cytokine levels in plasma samples from late stage EMF patients. Cytokine levels of Tumor Necrosis Factor (TNF)-α, Interferon (IFN)-γ, Interleukin (IL)-2, IL-4, IL-6, and IL-10 were assayed in plasma samples from 27 EMF patients and compared with those of healthy control subjects. All EMF patients displayed detectable plasma levels of at least one of the cytokines tested. We found that TNF-α, IL-6, IL-4, and IL-10 were each detected in at least 74% of tested sera, and plasma levels of IL-10, IL-4, and TNF-α were significantly higher than those of controls. Plasma levels of such cytokines positively correlated with each other.

**Conclusions/Significance:**

The mixed pro- and anti-inflammatory/Th2circulating cytokine profile in EMF is consistent with the presence of a persistent inflammatory stimulus. On the other hand, the detection of increased levels of TNF-α may be secondary to the cardiovascular involvement observed in these patients, whereas IL-4 and IL-10 may have been upregulated as a homeostatic mechanism to buffer both production and deleterious cardiovascular effects of pro-inflammatory cytokines. Further studies might establish whether these findings play a role in disease pathogenesis.

## Introduction

Tropical endomyocardial fibrosis (EMF) is a restrictive cardiomyopathy characterized by fibrous tissue deposition of the endomyocardium of one or both ventricles, associated with diastolic heart failure (HF), secondary valvular dysfunction, and atrial arrhythmias, such as atrial fibrillation (AF). The etiopathogenesis of EMF is still obscure [1]. Several factors involving immune mechanisms have been suggested to play a pathogenetic role, including infections, chronic helminthic infection-related hypereosinophilia, allergy, auto-immunity, and malnutrition. One of the major pathogenetic theories states that EMF could be considered a late effect of helminthic infection-induced eosinophil degranulation in the heart,due to its similarities with the eosinophilic endocarditis (EE) of Loeffler's syndrome [2]–[4]. At the late stage of the disease, the presence of a focal perivascular chronic inflammatory infiltrate deep within the endomyocardium, predominantly composed by lymphocytes and macrophages, with very rare eosinophils is consistent with a role of persistent immune-mediated inflammation [5]. Cytokines are key mediators of immunity, modulating the nature of the immune and inflammatory responses. Proinflammatory cytokines such as TNF-α and IL-6 have been found to be increased both in peripheral blood and heart tissue, in several cardiovascular (CV) diseases including HF [6]–[8] and have prognostic significance [9], [10].

Direct pathogenic effects of TNF-α include progressive cardiomyocyte apoptosis, adverse ventricular remodelling, left ventricular (LV) wall thinning and dilation, which have been observed in mice overexpressing TNF-α [11]. Anti-inflammatory cytokines such as IL-4 and IL-10 are associated with helminthiasis and eosinophilia [12] and a limited number of studies have reported the detection ofsuch cytokines in CV disorders [13], [14]. Several of the clinical features characteristic of EMF are associated themselves with increased levels of circulating cytokines [15]. Even though a persistent local inflammatory infiltrate is found in late-stage EMF, it is yet unknown whether such patients display systemic inflammatory activation.In order to assess whether there is systemic inflammation in the late stages of EMF, we assessed the circulating levels of pro- and anti-inflammatory/Th2 cytokines in EMF patients and controls.

## Methods

The protocol was approved by the Institutional Review Board of the University of São Paulo School of Medicine (Protocol number 0569/10), and written informed consent was obtained from all the subjects.

### Patient selection

We evaluated cytokine plasma levels of 27 EMF outpatients (24 female, 34.6±15.5 years) followed at the Cardiomyopathy Unit of the Heart Institute (InCor), University of São Paulo Medical School between 2004 and 2012, and 38 healthy blood donors (26 female, 33.9±12.0 years) from Pró-Sangue Foundation, as control subjects. Patients had undergone bidimensional Doppler echocardiography and gadolinium-enhanced magnetic resonance imaging. The major inclusion criteria included clinical signs suggestive of diastolic HF, apical obliteration of one or both ventricles [16], [17] and late gadolinium enhancement magnetic resonance imaging showing the typical pattern of fibrous tissue deposition [18]. There were no exclusion criteria.Nine (30%) patients hadbiventricular, seven (26%) right ventricular, and eleven (44%) left ventricular (LV) involvement. Patients came from lower socioeconomic strata, with poor housing and evidence of protein malnutrition during childhood. Twenty-one patients (78%) underwent surgical resection of endomyocardial fibrous tissue; gross anatomy and histopathology confirmed the diagnosis.

### Sample collection and cytokine quantification

EDTA-anticoagulated peripheral blood samples were collected from the patients and from healthy controls. Plasma samples were stored at −80°C until cytokine assays were performed, using the Th1/Th2 II human bead array (BD Biosciences), according to the manufacturer's recommendations. Samples were analyzed with the FACSCanto flow cytometer (BD Biosciences), and the FCAP Array software (BD Biosciences) was used for data analysis.

### Statistical Methods

Cytokine levels were compared among EMF patients and subject controls by using the Mann-Whitney *U* test. Correlations of data were analyzed by the non-parametric Spearman test. All analysis was performed with Graph Pad Prism version 5 software (GraphPad Software, La Jolla, California, USA). A *P* value<0.05 was considered statistically significant.

## Results

**Figure 1:**
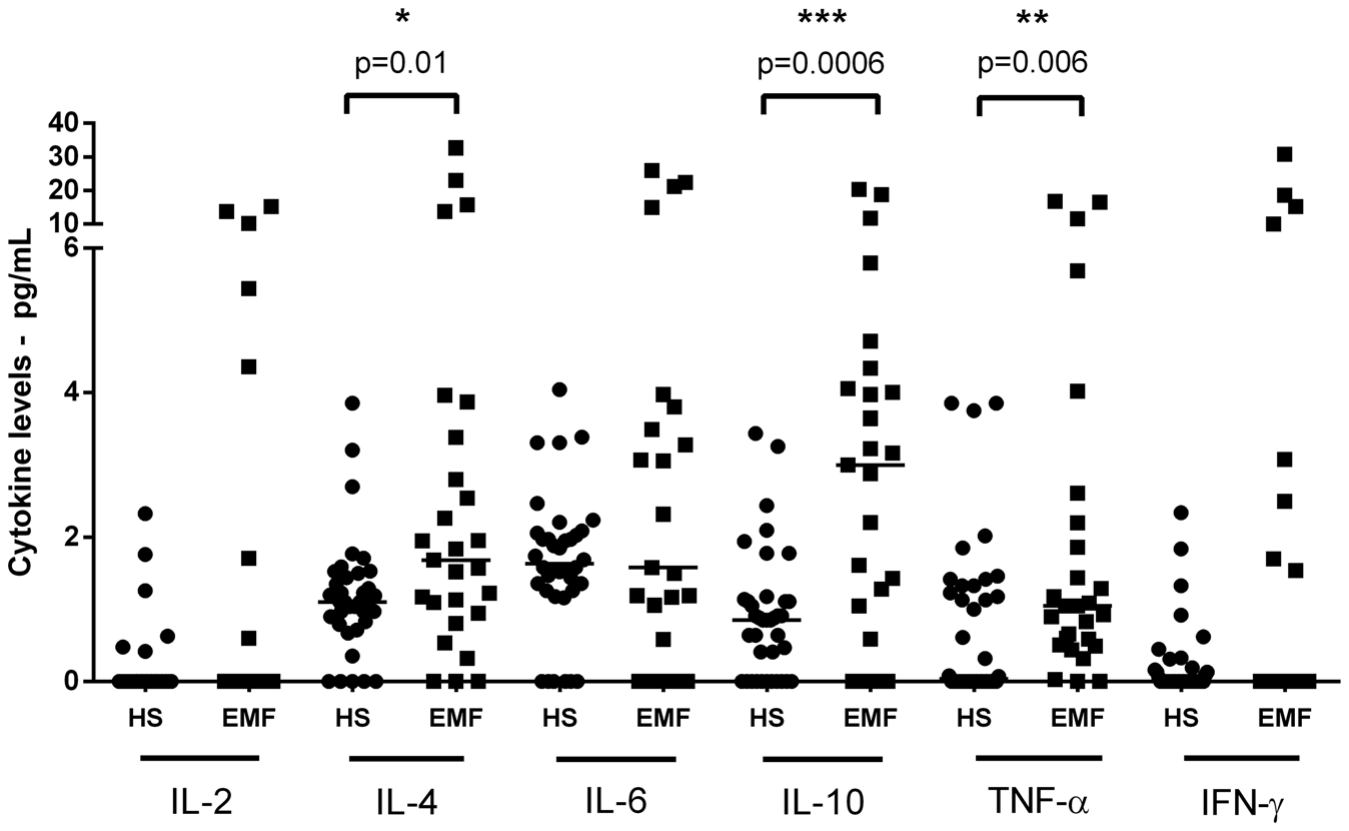
Cytokine plasma levels of endomyocardial fibrosis patients and healthy subjects. Dot plot represents cytokine levels (interleukin 2, 4, 6, 10, TNF-α and IFN-γ) from endomyocardial fibrosis patients (EMF) and healthy subjects (HS), evaluated by CBA. Horizontal lines indicate the median values. *Differences where *P*≤0.05 are indicated. (•Healthy Subjects;▪EMF patients).

**Figure 2:**
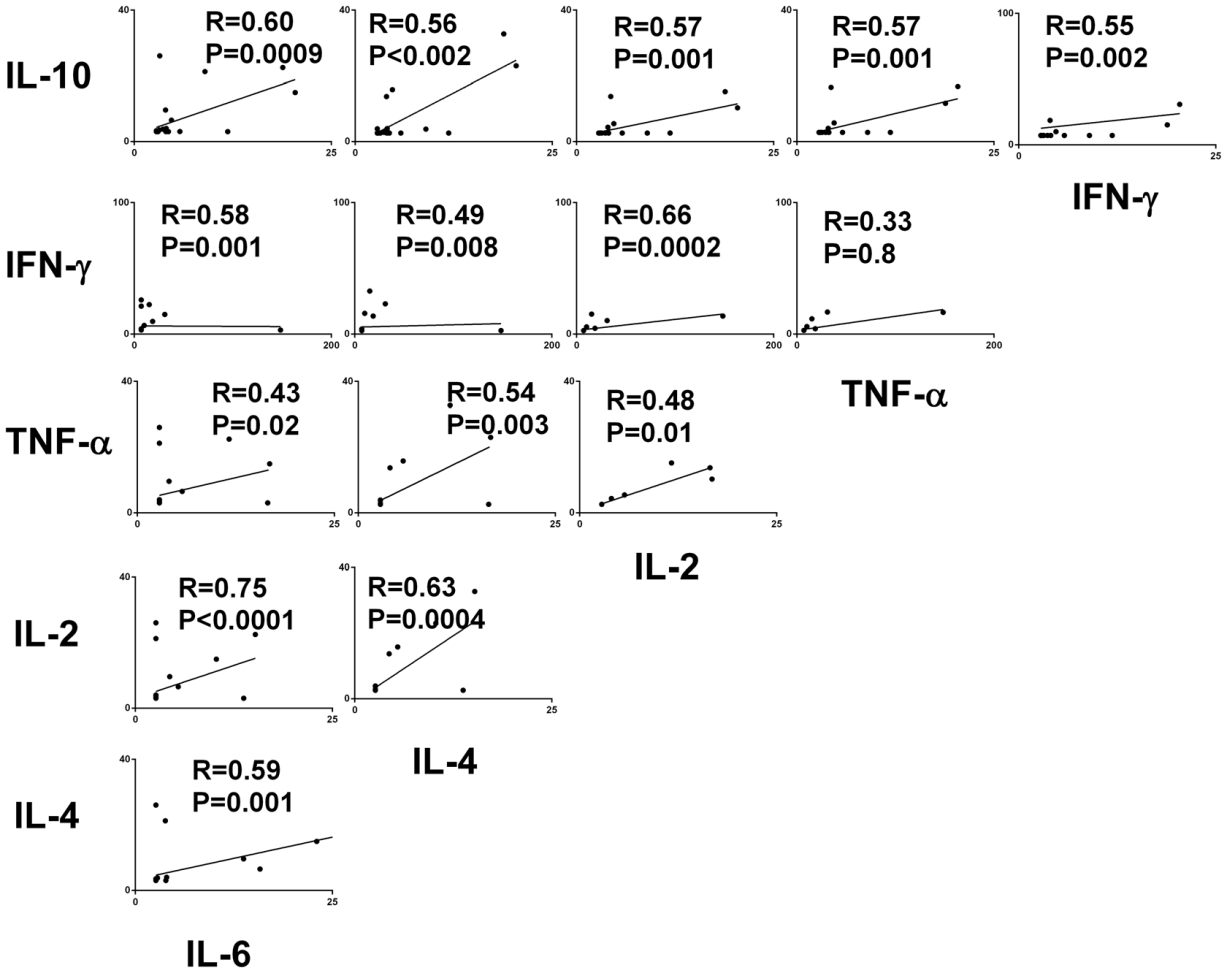
Correlation among cytokine plasma levels of endomyocardial fibrosis patients. Dot plots represent correlation between cytokine levels (interleukin 2, 4, 6, 10, TNF-α and IFN-γ) from endomyocardial fibrosis patients, evaluated by CBA.

EMF and healthy subjects were age and sexmatched (Table 1). Plasma samples from all 27 EMF patients examined in this study showed detectable levels of at least one of the assayed cytokines. Increased levels of interleukin (IL)-4 (4.51±7.79 pg/mL), IL-10 (4.11±5.27 pg/mL), and tumor necrosis factor alpha (TNF-α) (2.77±4.64 pg/mL) were detected in a high proportion of EMF patients (88.8%, 92.6%, and 77.7%, respectively) compared with healthy control samples (1.22±0.87 pg/mL - *P* = 0.001, 0.99±0.89 pg/mL- *P* = 0.0001 and 0.94±0.24 pg/mL - *P* = 0.006, respectively - Mann Whitney U Test) - (Figure 1 and 2). There was no significant difference in IL-6, IFN- γ and IL-2 levels compared with control samples. Interestingly, circulating IL-4 was present in 20/21 samples in which IL-10 was detectable. In addition, a positive correlation was observed between all cytokine levels in EMF patients, with the exception of TNF-α and IFN- γ (*P* = 0.08; Figure 2).

**Table 1:** Demographic and echocardiographic data from EMF patients and healthy subjects.

Variable	EMF	HS
**Gender (Male/Female)**	3/24	12/26
**Age (years - Male/Female)**	34.6±15.5	33.9±12
**Bilateral/RV/LV EMF (%)**	49.5±13.3	53.9±12,9
Mitral regurgitation (%) *	30/26/44	NA
Tricuspid regurgitation (%)*	55.5%/26%/7.4%	NA
Diastolic dysfunction grade (%)**	37%/18.5%/7.4%	NA
**Systolic dysfunction (%)**	60,90%	NA
**AF (%)**	21.4	NA

NA – Not applicable; AF – Atrial Fibrillation; (Percentage of EMF patients with the conditionregistered in the medical records); Systolic dysfunction: Ejection Fraction <55%; *Valvar regurgitation level: mild, moderate, and severe, respectively; Diastolic dysfunction: grades mild, moderate, and severe ** It was not possible to evaluate diastolic function in 4 patients, due to the presence of pacemaker or bioprosthetic heart valve.

## Discussion

Our study shows that EMF patient shave elevated plasma levels of pro- and anti-inflammatory cytokines, especially TNF-α, IL-4, and IL-10, which were significantly higher than those in healthy subjects. The correlation between levels of pro- and anti-inflammatory/Th2 cytokines may suggest that either the stimuli that induce both kinds of cytokines are the same, or that IL-10 and IL-4 may have a regulatory role and control the levels and deleterious effects of proinflammatory cytokines.

Multiple cardiovascular disorders are associated with increased levels of circulating pro-inflammatory cytokines, especially TNF-α and IL-6, which are related to a common cytokine profile found in acute and chronic HF patients independently of etiology [19], [20]. Mann proposed that acute increased levels of IL-6, TNF-α and IL-1 could be an adaptive response for cardiac injury, with cardioprotective effects, whereas the chronic elevation of these mediators could be a maladaptive response and promote cardiac decompensation [21]. However, the underlying cause for the presence of the inflammatory response in different CV diseases is still under investigation [22]. It has been suggested that the predominant mechanism of upregulation of TNF-α production is secondary to advanced heart failure itself, such as low cardiac output and intestinal bacterial translocation [23].

In addition, a limited number of reports on HF have found increased plasma levels of the other cytokines detected in our study (IL-10, IL-4, and to a lesser extent IFN- γ and IL-2) [13], [24]–[26]. It is thus possible that the triggers of cytokine production in EMF are similar to those found in HF with other causes, such as hypertensive or idiopathic dilated cardiomyopathy. On the other hand, our findings that late-stage EMF patients display increased IL-4 and IL-10 levels are also consistent with the observed early eosinophilia and helminthic infections in EMF, once this type of infection is usually associated with increased levels of these cytokines [27]–[30]. Whichever the stimulus for cytokine production may be, results suggest a possible relevance of a persisting Th2 (IL-4 and IL-10) cytokine-driven immune mechanism in the pathogenesis of EMF. Significantly, IL-10 is an anti-inflammatory cytokine that may reduce TNF-α production, which may be clinically significant in the pathogenesis of HF [31]. In our study, the almost universal co-detection of inflammatory and anti-inflammatory cytokines, as well as the correlation between their levels is consistent with such an antagonistic effect. On the other hand, along with its known anti-inflammatory effects, long-term overexpression of IL-10 has been associated with lung fibrosis [32].

Although blood eosinophilia (BE) has been reported in EMF cases [29], less than 40% of our patients displayed BE (≥500 eosinophils/mm^3^) during follow-up in the chronic phase of this disease. Our data are consistent with those reported by Patel and associates (1977) [33], who observed that the absolute BE in African EMF patients was similar to that of healthy control subjects. In a recent study from a similar cohort of patients in our Hospital, Iglezias et al. [5] found no eosinophils in the heart lesions of EMF patients. Together, these results suggest that neither blood nor endocardial eosinophilia are essential components of late-stage EMF. Our data are consistent with the hypothesis that early helminthic infestation could cause a waning eosinophilia that might be involved in initial heart damage in EMF pathogenesis, and along with a long-lasting Th2 response whose pathogenic or protective potential is yet unclear. However, we cannot exclude that the anti-inflamatory cytokine levels are raised as a homeostatic mechanism to buffer both production and effects of pro-inflammatory cytokines. Although persistent IL-10 production may lead to lung fibrosis [33], it is unknown whether the cytokine could accelerate fibrous tissue deposition in the endomyocardium of EMF patients. One limitation of this study is the lack of information about helminthic infection status in our patient group.

In summary, we have shown for the first time that late-stage EMF patients display detectable plasma levels of a mixed pro- and anti-inflammatory/Th2 cytokine profile, predominantly composed of TNF-α, IL-10 and IL-4 levels. The finding of such a mixed cytokine profile may either reflect the multiple cardiovascular disorders also experienced by EMF patients, or indicate a common persistent stimulus for production of both pro- and anti-inflammatory/Th2 cytokines. On the other hand, anti-inflammatory/Th2 cytokines IL-4 and IL-10 may either be upregulated by previous helminthic infection, or as a homeostatic mechanism to buffer both production and effects of pro-inflammatory cytokines. This antagonism is consistent with the almost universal co-detection of inflammatory and anti-inflammatory cytokines in EMF plasma samples, as well as the positive correlation between their plasma levels. Further studies may allow the identification of the stimulus for chronic cytokine production, and to establish whether cytokines play a role in pathogenesis or have a prognostic value for rates of disease progression or post-surgical follow-up.
